# At-wavelength characterization of X-ray wavefronts in Bragg diffraction from crystals

**DOI:** 10.1107/S1600577523007531

**Published:** 2023-10-10

**Authors:** Xianbo Shi, Zhi Qiao, Paresh Pradhan, Peifan Liu, Lahsen Assoufid, Kwang-Je Kim, Yuri Shvyd’ko

**Affiliations:** a Argonne National Laboratory, 9700 South Cass Avenue, Lemont, IL 60439, USA; Tohoku University, Japan

**Keywords:** wavefront sensing, crystal diffraction, coded mask, phase error, at-wavelength metrology

## Abstract

The advent of next-generation synchrotron radiation sources and X-ray free-electron lasers calls for high-quality Bragg-diffraction crystal optics to preserve the X-ray beam coherence and wavefront. This requirement brings new challenges in characterizing crystals in Bragg diffraction in terms of Bragg-plane height errors and wavefront phase distortions. Here, a quantitative methodology to characterize crystal optics using a state-of-the-art at-wavelength wavefront sensing technique and statistical analysis is proposed. The method was tested at the 1-BM-B optics testing beamline at the Advanced Photon Source for measuring silicon and diamond crystals in a self-referencing single-crystal mode and an absolute double-crystal mode. The phase error sensitivity of the technique is demonstrated to be at the λ/100 level required by most applications, such as the characterization of diamond crystals for cavity-based X-ray free-electron lasers.

## Abstract 2

A quantitative methodology utilizing at-wavelength wavefront sensing is developed for characterizing X-ray wavefronts in Bragg diffraction from high-quality crystal optics for next-generation synchrotron radiation sources and X-ray free-electron lasers.

## 1. Introduction

X-ray Bragg diffraction crystal optics play essential roles in modern X-ray light sources as monochromators (Matsushita & Hashizume, 1983 ▸; Toellner, 2000 ▸; Shvyd’ko, 2004 ▸), spectral analyzers (Masciovecchio *et al.*, 1996 ▸; Sinn, 2001 ▸; Shvyd’ko, 2004 ▸; Verbeni *et al.*, 2005 ▸; Baron, 2016 ▸) and beam splitters for such applications as beam-multiplexing monochromators (Als-Nielsen *et al.*, 1994 ▸; Stoupin *et al.*, 2014 ▸; Zhu *et al.*, 2014 ▸), split-and-delay X-ray photon correlation spectrometers (Roseker *et al.*, 2011 ▸; Osaka *et al.*, 2013 ▸; Stetsko *et al.*, 2013 ▸; Hirano *et al.*, 2018 ▸) and X-ray cavity output couplers (Shvyd’ko, 2019 ▸). As the next-generation synchrotron radiation sources and free-electron lasers (FELs) are either operating or being built, crystals with ‘perfect’ qualities are in high demand. For these sources, the X-ray diffraction crystals need to be perfect in their structure, cut and, more importantly, surface finishing. An accurate and strain-free surface polishing becomes the key to preserving the high-quality X-ray beam wavefront emitted by these new sources.

Crystal quality can be quantified in several ways, and the choice of method can depend on the application. The traditional criterion is the Bragg-plane slope error, which is most appropriate for monochromator applications. Crystal-based monochromators are widely used for synchrotron radiation sources to provide X-ray beams with a narrow spectral bandwidth. For example, a perfect Si(111) crystal with flat Bragg planes has an intrinsic relative bandwidth of Δ*E*/*E* ≃ 10^−4^ and an angular width of Δθ_B_ = 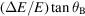 , where θ_B_ is the diffraction angle defined by Bragg’s law and the photon energy *E*. The Bragg-plane deformation, introduced by strain, will broaden the bandwidth and distort the wavefront. Traditionally, Bragg-plane slope error is the main parameter used to define the crystal quality, with a general requirement that this error be much less than the intrinsic Bragg reflection angular widths. The primary diagnostic technique is Bragg reflection (rocking) curve topography imaging (RCI) (Lübbert *et al.*, 2000 ▸), which directly measures the local rocking curves and Bragg-plane slope errors with a micrometer-level spatial resolution. For high-quality silicon and diamond single crystals, Bragg-plane slope errors as small as ∼100 nrad RMS (root mean square) — much smaller than most of the Bragg reflection widths used in practice — were found to be feasible (Pradhan *et al.*, 2020 ▸).

A different approach is warranted when X-ray Bragg diffraction flat crystal optics are combined with X-ray focusing optics; for example, in X-ray spectrographs (Kohn *et al.*, 2009 ▸; Shvyd’ko *et al.*, 2014 ▸; Shvyd’ko, 2015 ▸, 2016 ▸; Chumakov *et al.*, 2019 ▸; Kim *et al.*, 2018 ▸; Bertinshaw *et al.*, 2021 ▸). In this case, information on wavefront distortions upon Bragg reflection becomes more appropriate for achieving perfect focusing or imaging. Here, the crystal quality is better described by the Bragg-plane height error, which is directly related to the wavefront phase error. For the wavefront to be regarded as diffraction-limited, based on the Maréchal criterion (Maréchal, 1947 ▸), the RMS wavefront error must be below λ/14, with λ being the X-ray wavelength. In extreme cases, the limit must be set much lower; for example, in X-ray cavities of cavity-based X-ray free-electron lasers (CBXFEL), which have multiple low-loss wavefront-preserving flat Bragg-reflecting crystal mirrors and aberration-free focusing elements that store and circulate X-ray beams (Kim *et al.*, 2008 ▸, 2012 ▸; Kim & Shvyd’ko, 2009 ▸; Lindberg *et al.*, 2011 ▸; Dai *et al.*, 2012 ▸; Freund *et al.*, 2019 ▸; Marcus *et al.*, 2020 ▸). In this sensitive case, the wavefront error induced by each crystal reflection is required to be as small as λ/100. This super-high specification brings not only many crystal fabrication challenges but also necessitates high-sensitivity wavefront sensing techniques. In addition, high-quality diamond crystals are of particular interest in many applications, such as CBXFEL, due to their high thermal conductivity, mechanical stiffness, X-ray transparency and other unique features (Shvyd’ko *et al.*, 2017 ▸).

Meeting these challenges requires the characterization of X-ray optics at the operational wavelength. This type of characterization, called at-wavelength metrology, is the ultimate approach for determining optics quality and its effects on the wavefront. Several such techniques exist, though none are fully adequate for the high sensitivity required for applications such as CBXFELs. Existing advanced at-wavelength wavefront sensing techniques are based on either grating interferometry (Weitkamp *et al.*, 2005 ▸; Wang *et al.*, 2011 ▸; Grizolli *et al.*, 2017 ▸) or near-field speckle tracking (Berujon *et al.*, 2020*a* ▸,*b* ▸). A recently developed coded-mask method (Qiao *et al.*, 2021*a* ▸,*b* ▸, 2022 ▸) combines the advantages of both approaches with superior resolution and sensitivity. It has been successfully demonstrated for applications in at-wavelength metrology and phase-contrast imaging in standard transmission geometry. However, using this method for crystal characterization is challenging because of the diffraction geometry, especially for ultra-high-quality crystals that produce only small wavefront distortions. In this work, we study different measurement geometries and propose the optimal approach for crystal wavefront characterization using a coded-mask method. The approach was demonstrated in experiments carried out at the 1-BM-B optics testing beamline (Macrander *et al.*, 2016 ▸) at the Advanced Photon Source (APS), Argonne, USA. Characterization results of high-quality silicon and diamond crystals are presented, along with statistical analysis over the entire crystal surface showing the distribution of the local Bragg-plane height error and the effects of the error on wavefront quality.

## 2. Method

### 2.1. Coded-mask-based wavefront sensing

The critical component of any near-field speckle-tracking wavefront sensing technique is a speckle generator to introduce a random speckle pattern in the beam images. Traditionally, a piece of sandpaper or a membrane filter is used as the speckle generator, but such materials generate complex pattern structures that are not necessarily optimal for the sample and beam condition being tested. By contrast, we employ a predesigned random-phase mask to accommodate different test samples and beam conditions and to produce ultra-high-contrast speckle images. The high contrast in these images allows high resolution and speed to be achieved by using advanced phase-retrieval algorithms, such as maximum-likelihood optimization (Qiao *et al.*, 2021*a* ▸) and machine learning (Qiao *et al.*, 2022 ▸).

The coded mask is usually a binary mask having randomly distributed pixels, with each pixel configured to have a 50% probability of being a high-phase pixel or a low-phase pixel, wherein a high-phase pixel alters the wavefront phase by a higher amount relative to the low-phase pixel. This relative difference between the high-phase and low-phase pixels’ phase shift values is selected from 0 to 2π (typically close to π for the highest contrast). The pitch size of the mask pixel (usually a few micrometers) is chosen to be as small as possible while still being resolvable by the imaging camera.

At-wavelength wavefront metrology of an optic usually involves collecting two data sets, a ‘sample’ data set (with the optic in the beam path) and a ‘reference’ data set (without the optic), both having the speckle generator (*e.g.* coded mask) in the beam path. A data set can be a single image (Qiao *et al.*, 2021*a* ▸) or an array of images taken in scanning mode (Qiao *et al.*, 2021*b* ▸). By comparing the two data sets, the local displacement, δ(*x*, *y*), of the speckle pattern can be obtained, where (*x*, *y*) is in the transverse plane perpendicular to the wavefront propagation direction, *z*. Then the local beam deflection angle vector α(*x*, *y*) caused by the optic can be extracted as 

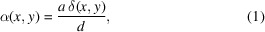

where *a* is a geometric scale factor taking into account the geometric magnification of the beam. The beam propagation distance *d* is the sample-to-detector distance if the sample is downstream of the coded mask or the mask-to-detector distance if the sample is upstream.

The gradient of the wavefront phase shift, ∇ϕ(*x*, *y*), is then a function of α(*x*, *y*), given by 

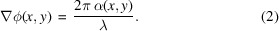

The map of wavefront phase shifts resulting from the insertion of an optic, ϕ(*x*, *y*), can be obtained by integrating the phase shift gradients in the two orthogonal transverse directions, ∂ϕ/∂*x* and ∂ϕ/∂*y*.

**Figure 1:**
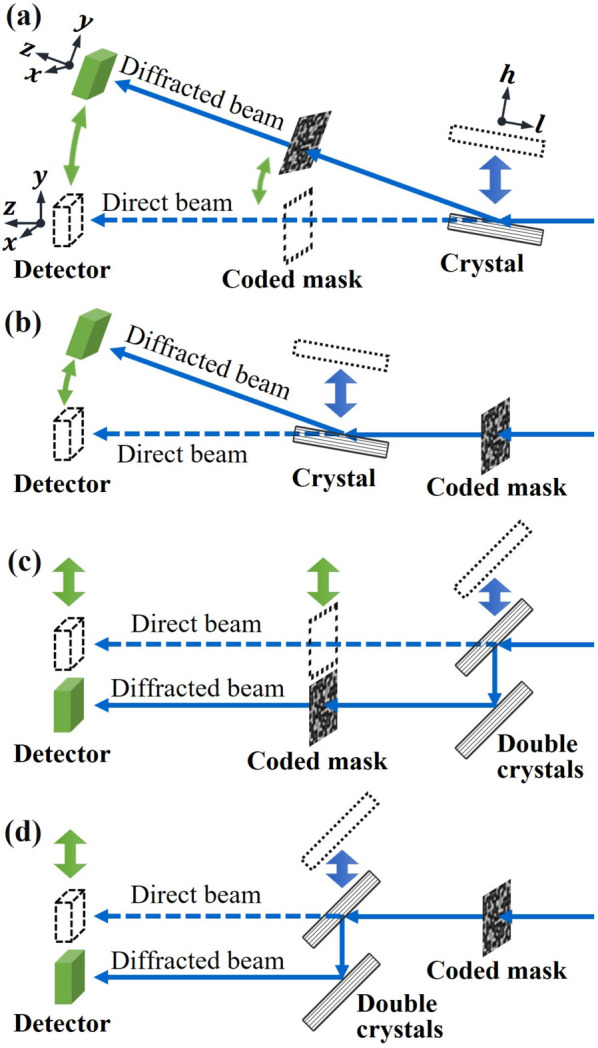
Schematics for measuring wavefront distortions upon Bragg reflection from one crystal (*a* and *b*) or from a nondispersive double-crystal arrangement (*c* and *d*) with the coded mask upstream (*a* and *c*) or downstream (*b* and *d*) of the crystals. The dashed lines show the direct beam path when the crystal(s), mask and detector move to the dotted positions.

Next, we aim to connect the phase shift ϕ(*x*, *y*) in the transverse plane (*x*, *y*), which is perpendicular to the beam propagation direction *z*, to the crystal’s height error *h*(*x*, *l*) in its surface coordinate (*x*, *l*). In this context, the crystal is placed in a symmetric Bragg diffraction geometry where the diffraction plane is the (*y*, *z*) plane. All coordinates are illustrated in Fig. 1 ▸(*a*). The measured phase shift ϕ(*x*, *y*) can be simply projected to the (*x*, *l*) plane as 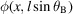 . Considering that the phase shift correlates with the path length difference 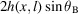 by a factor of 2π/λ, this leads to 

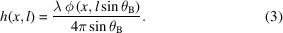




### 2.2. Geometry

Four possible geometries for measuring wavefront distortions upon Bragg reflection from crystals are shown in Fig. 1 ▸, with the direct beam recorded as the reference image and the diffracted beam as the sample image.

In Fig. 1 ▸(*a*), the coded mask is positioned downstream of the crystal. The diffracted beam from the crystal, upon reaching the detector, is flipped upside down in the diffraction plane compared with the direct beam image. However, this inversion does not occur for the detected mask pattern. As a result, the observed speckle displacements encapsulate not only the crystal-induced distortion but also the phase information from the source beam flipping. This intermingling of effects prevents us from isolating and analyzing the sole impact of the crystal.

In Fig. 1 ▸(*b*), with the coded mask placed upstream of the crystal, both the beam and the speckle pattern are inverted in the diffracted beam on the detector. It is possible to manually revert this diffraction image and compare it with the direct image. However, this procedure assumes the detector retains identical properties upon inversion. At first glance, this assumption seems plausible, but experimental outcomes indicate suboptimal results. Beyond the detector’s inherent imperfections, which could potentially be corrected through calibration, additional challenges lie in maintaining detector stability and accurate alignment along the 2θ_B_ diffraction arm. We believe these aspects significantly contribute to the experimental difficulties.

There is another fundamental problem when the coded mask is placed downstream of a one-crystal or a double-crystal set, as shown in Figs. 1 ▸(*a*) and 1 ▸(*c*), respectively. If a crystal has a small miscut angle (or different miscut angles for the two crystals), the direct and diffracted beams will have different sizes, thus covering different areas of the mask pattern. This discrepancy makes speckle tracking in the nonoverlapping areas impossible.

Given these considerations, the geometry in Fig. 1 ▸(*d*) is our selected arrangement for measuring wavefront distortions in Bragg diffraction. The nondispersive double-crystal diffraction geometry ensures parallelism between the direct and diffracted beams. Keeping a small offset between the two crystals can also reduce the mechanical instability and measurement errors due to the motion of the detector.

However, the geometry in Fig. 1 ▸(*d*) is not without issues. Of course, it allows the characterization only of the combination of two crystals. Another challenge is that the speckle pattern, *B*_*H*_, is blurred by 

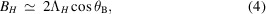

in the diffraction plane because Bragg diffraction with diffraction vector *H* takes place within a crystal surface layer of thickness, the so-called extinction effect, where Λ_*H*_ is the extinction length that is typically ∼1–100 µm depending on the Bragg reflection. This one-dimensional blurring effect makes the speckle tracking between the direct-beam and diffracted-beam images challenging, because the blurring causes large phase-detection errors, especially at high spatial frequency. Nevertheless, many experimental tests have been carried out, and the geometry in Fig. 1 ▸(*d*) has proved effective and provides better performance than the other geometries.

To characterize the quality of just one of the crystals in the double-crystal arrangement, we propose a self-referencing method based on the geometry in Fig. 1 ▸(*d*). In this method, one of the crystals (usually the downstream one) is moved laterally in its surface plane so that diffracted beam images are measured from different areas of the crystal surface. A relative phase shift map extracted from any two of these images (one as ‘reference’ and the other as ‘sample’ data sets) contains combined effects of the two areas of the moving crystal but excludes the effects of the nonmoving one. Since no direct beam is involved, the blurring effects due to nonzero extinction length are present for both images and are thus canceled in the analysis. Details on how to interpret the results from these measurement schemes are presented in Section 4.1. In Section 4.2, we show the wavefront error measurements of the double-crystal assembly in absolute mode using Fig. 1 ▸(*d*) geometry. In this instance, the ‘reference’ dataset is the direct beam image without crystals, while the ‘sample’ dataset is the diffracted image after both crystals.

## 3. Experiment

**Figure 2:**
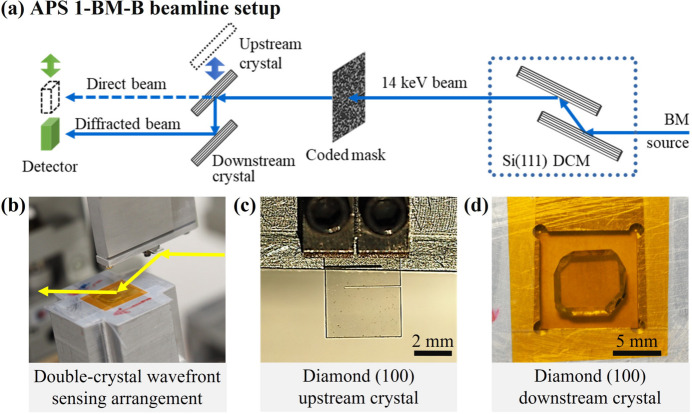
(*a*) Schematic of the wavefront sensing setup at APS beamline 1-BM-B, and photographs of (*b*) a double-crystal arrangement, (*c*) the upstream crystal — a diamond (100) crystal in the 400 reflection with strain-relief cuts — and (*d*) the downstream crystal — a diamond (100) crystal in the 400 reflection in the sample mount.

At-wavelength wavefront sensing of crystal diffraction was carried out at the 1-BM-B optics testing beamline at the APS with the setup shown in Fig. 2 ▸. X-rays with a photon energy of 14 keV were selected by a Si(111) double-crystal monochromator (DCM) placed at 27.5 m downstream of the bending-magnet source. The double-crystal wavefront sensing arrangement shown in Fig. 2 ▸(*b*) was situated 31.5 m downstream of the source.

The almost flawless single crystals of silicon and diamond used in the present studies were previously characterized by RCI (Pradhan *et al.*, 2020 ▸). The crystals exhibit very small Bragg-plane slope errors of ≲0.1 µrad (RMS) in selected regions of interest of ∼1 mm^2^. Special care was taken to mount crystals strain-free to avoid artificial distortions.

The upstream crystal, serving as a conditioning crystal in the double-crystal setup, was a high-quality diamond single crystal in the (100) orientation of type IIa. It was grown under high pressure and high temperature (HPHT) conditions at the Technological Institute for Superhard and Novel Carbon Materials (TISNCM) in Moscow, Russia (Blank *et al.*, 2007 ▸; Polyakov *et al.*, 2011 ▸). The diamond crystal was set into the 400 Bragg diffraction with a Bragg angle θ_400_ = 29.77° and an extinction length of Λ_400_ = 3.6 µm. The 440 µm-thick diamond crystal was furnished with two strain-relief cuts (Pradhan *et al.*, 2020 ▸), as shown in Fig. 2 ▸(*c*), to prevent the clamping strain from propagating to the working area.

The downstream crystal in the double-crystal setup is the test crystal that is under study in self-referencing mode. We characterized two crystals in the downstream test position. In the first test, we used a high-quality Si crystal to assess the sensitivity of the method to wavefront phase distortions and Bragg-plane errors. The crystal was set into the 531 Bragg reflection with Bragg angle θ_531_ = 28.84°, extinction length Λ_531_ = 5.3 µm, and the entrance surface of the crystal cut parallel to the (531) plane. The 531 Bragg reflection was specifically selected to match as closely as possible the Bragg angle of the 400 Bragg reflection of the upstream diamond crystal; this choice means the beam reflected from the downstream crystal will be close to parallel to the beam incident on the upstream crystal.

In the second test, we applied this technique to measure Bragg-plane height errors and wavefront distortions in a second high-quality type IIa HPHT diamond crystal made by TISNCM. We chose the best diamond crystal in the (100) orientation that was available to us. It was previously characterized with RCI (Pradhan *et al.*, 2020 ▸) and denoted in that study as VB4. Like the upstream diamond crystal, this VB4 crystal has also been shown to exhibit a near 100% Bragg reflectivity of X-rays (Shvyd’ko *et al.*, 2011 ▸). Previous studies using RCI have shown that the Bragg-plane slope errors of this VB4 crystal are only slightly larger than in the Si(531) crystal (Pradhan *et al.*, 2020 ▸). The diamond crystal lay freely in a recess of an aluminium crystal holder covered with a thin Kapton foil, as shown in Fig. 2 ▸(*d*). The phase error due to the Kapton foil was tested separately; the effects were negligible.

The speckle generator was a coded binary phase mask with a pitch size of 10 µm. The coded mask was inserted into the X-ray beam about 130 mm upstream of the double-crystal setup. The pitch size was chosen to make the speckle size as small as possible for the best spatial resolution but large enough to overcome the speckle-blurring effects mentioned in Section 2.2. The detector system consisted of a 100 µm-thick LuAG:Ce scintillator, a 10× objective lens, and an Andor Neo sCMOS camera; it was placed at a crystal-to-detector distance of *d* = 893 mm. The detector system has an effective pixel size of 0.65 µm and an estimated spatial resolution of 2.2 µm (Koch *et al.*, 1998 ▸).

## 4. Results

### 4.1. Wavefront error measurement in self-referencing mode

**Figure 3:**
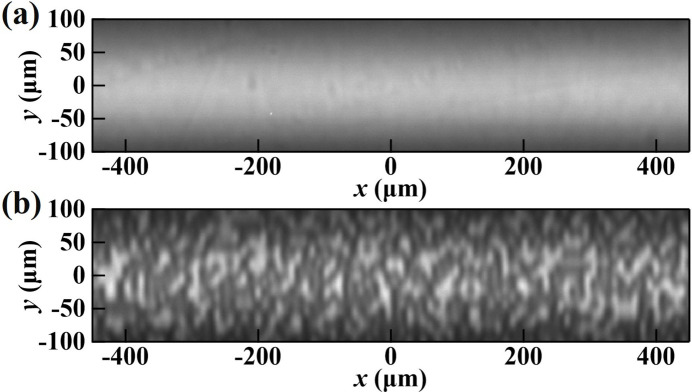
Diffraction images of the Si(531) crystal taken in area *A*_0_ (*a*) without and (*b*) with the coded mask in the beam path.

The high-quality Si(531) crystal was used in the downstream test position of the double-crystal arrangement to demonstrate the sensitivity of the self-referencing measurement and data analysis procedures. Recall that in the self-referencing mode two areas of the test crystal are examined, designated here *A*_0_ and *A*_1_, yielding ‘reference’ and ‘sample’ images (data sets), respectively. First, we screened the whole Si(531) crystal and identified a suitable reference area, *A*_0_, that was free of any visible defects in the X-ray diffraction image [Fig. 3 ▸(*a*)]. The vertical size of the diffraction image [Fig. 3 ▸(*a*)] is limited by the angular acceptance of the high-order Bragg reflections from the crystals and the dispersive geometry relative to the Si(111) Bragg-reflecting crystals of the beamline DCM. Figure 3 ▸(*b*) shows the high-contrast speckle pattern generated by the coded mask at area *A*_0_; this image was used as the ‘reference’ image for the wavefront error analysis. Note that the speckle pattern is smeared in the vertical direction, with lower contrast than in the horizontal direction, due to the extinction effect in Bragg diffraction, as presented by equation (4) in Section 2.2.

**Figure 4:**
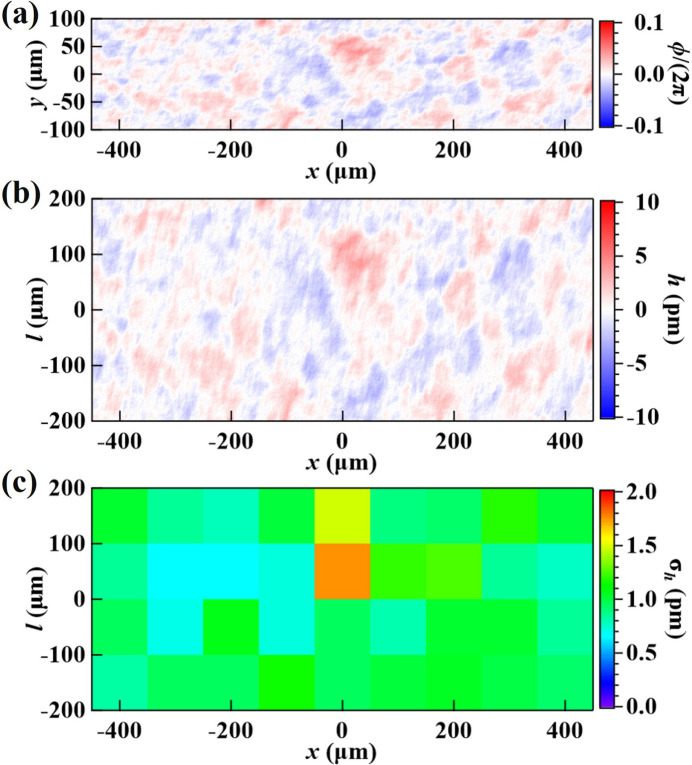
(*a*) Phase difference profile, ϕ(*x*, *y*), between the diffraction images taken in the ‘reference’ and ‘sample’ areas on the Si(531) test crystal. (*b*) Relative Bragg-plane height error profile, *h*(*x*, *l*), between those two areas. Note that the wavefront coordinate *y* and the crystal surface coordinate *l* in the diffraction plane have the linear relationship *y* = 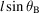 . (*c*) RMS height errors, σ_h_, calculated within 100 m × 100 m (*x* × *l*) subareas extracted from plot (*b*).

To complete the self-reference measurement, we moved a different area of the Si(531) crystal, *A*_1_, laterally into the same field of view and obtained the ‘sample’ diffraction image, taken with the same phase mask area in the beam. Using the ‘reference’ and ‘sample’ images, we reconstructed the relative wavefront phase difference profile using a wavelet-transform-based speckle vector tracking method (Qiao *et al.*, 2020 ▸); the result is shown in Fig. 4 ▸(*a*). Hereafter, the RMS deviation of the relative wavefront phase ϕ calculated over some area is referred to as the RMS phase error, σ_ϕ_. The RMS height error, σ_h_, is similarly defined and related to σ_ϕ_ via equation (3). The RMS phase error over the entire plotted area in Fig. 4 ▸ is σ_ϕ_ = 0.073 (or λ/86), which corresponds to a Strehl ratio (a measure of the quality of optical image formation) of *S* = 0.995, following the equation 

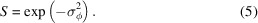

The corresponding crystal Bragg-plane height error, extracted using equation (3), is shown in Fig. 4 ▸(*b*).

In some applications, such as the multicrystal X-ray cavities of CBXFELs, the beam footprint on the crystal surface spans only a few hundred micrometers. In such cases, it is beneficial to characterize crystals within subareas of that size. Figure 4 ▸(*c*) shows the RMS height errors (σ_h_) within 100 µm × 100 µm (*x* × *l*) subareas. The color plot can assist in finding the best local areas on the crystal surface. The best subarea has the lowest RMS Bragg-plane height error of σ_h_ = 0.66 pm, corresponding to an RMS phase error of σ_ϕ_ = 0.045 (or λ/139) and a Strehl ratio of 0.998. This result demonstrates not only that the crystal is of super-high crystal quality but also that this wavefront sensing technique has a very high phase sensitivity, beyond λ/100.

**Figure 5:**
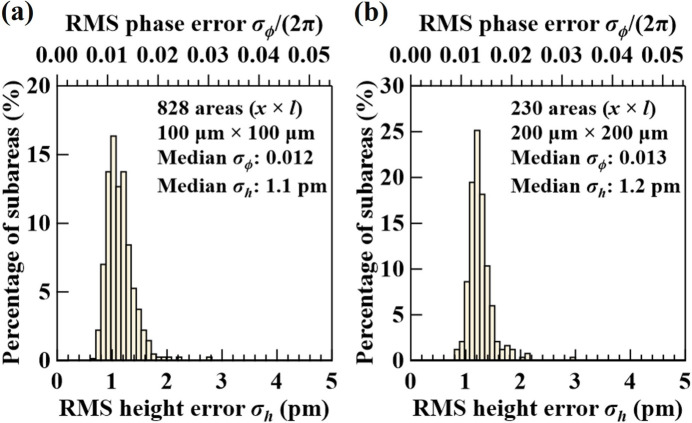
Histograms of the measured RMS height errors (bottom axis) and RMS phase errors (top axis) of all subareas with size (*x* × *l*) 100 m × 100 m (*a*) and 200 m × 200 m (*b*) over the entire Si(531) crystal.

Statistical analysis of subareas over the entire crystal surface can provide a quantitative measure of the overall crystal quality. For example, Figs. 5 ▸(*a*) and 5 ▸(*b*) show the statistical distribution of the RMS height errors and the corresponding RMS phase errors of all subareas with a size (*x* × *l*) of 100 µm × 100 µm and 200 µm × 200 µm, respectively, over the entire crystal. The median (value separating the higher half from the lower half) error across all subareas is taken to represent the overall quality of the crystal. We use the median instead of the average error to avoid contributions of a few extremely large or small values. A smaller median error value indicates better crystal quality, and a narrower error distribution indicates better uniformity of the crystal Bragg planes. Comparing results in Figs. 5 ▸(*a*) and 5 ▸(*b*), the median error values increase only slightly (<10%) going from 100 µm × 100 µm to 200 µm × 200 µm areas; it also indicates high homogeneity of the crystal quality.

**Figure 6:**
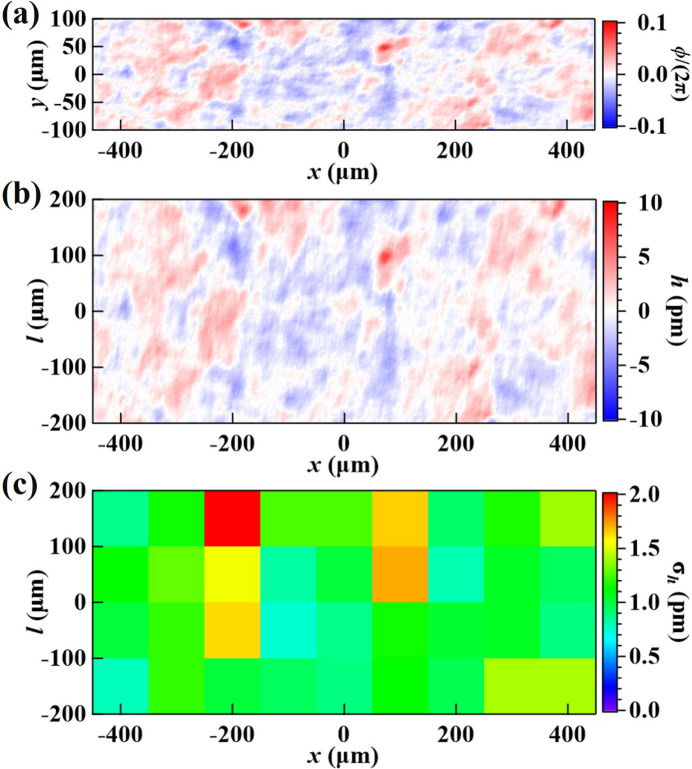
Same as Fig. 4 ▸ but showing results for the diamond (400) test crystal, VB4.

Using the same setup, we characterized the VB4 diamond (400) crystal in the downstream test position of the double-crystal arrangement; results of the wavefront measurements are shown in Fig. 6 ▸. The RMS phase error over the entire plotted area [Fig. 6 ▸(*a*)] is σ_ϕ_ = 0.098 (or λ/64) and the RMS Bragg-plane height error [Fig. 6 ▸(*b*)] is 1.39 pm. The best subarea [Fig. 6 ▸(*c*)] has an RMS Bragg-plane height error of 0.74 pm, corresponding to an RMS phase error of σ_ϕ_ = 0.050 (or λ/120) and a Strehl ratio of 0.997.

**Figure 7:**
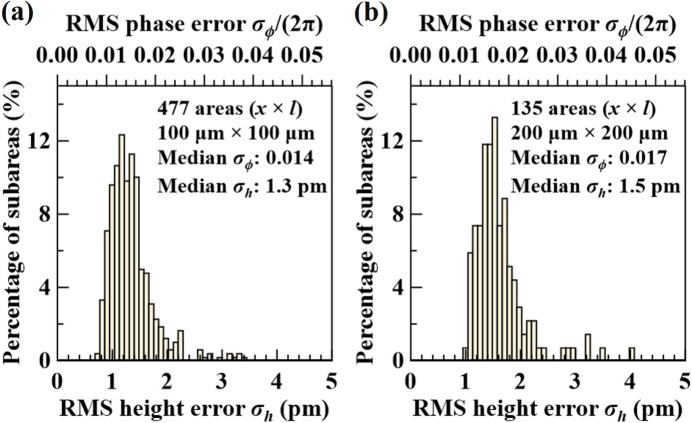
Same as Fig. 5 ▸ but showing results for the VB4 diamond (400) crystal.

Similarly, the histograms of the RMS phase errors of all subareas over the entire VB4 diamond (400) crystal are shown in Fig. 7 ▸. The quality of the VB4 diamond crystal is comparable with that of the Si(531) crystal (see Figs. 4 ▸ and 5 ▸), albeit not quite as high, which agrees with the conclusion of Pradhan *et al.* (2020 ▸). In particular, although the median RMS height error over all subareas is similar — 1.2 pm for Si(531) and 1.5 pm for C(400) — the width of the σ_h_ distribution for the 200 µm × 200 µm areas is a factor of two larger in the diamond crystal, indicating that, compared with the silicon crystal, the quality of this diamond crystal is less homogeneous.

### 4.2. Wavefront error measurements in absolute mode

We use the term absolute mode to refer to the geometry in Fig. 1 ▸(*d*), where the wavefront distortions of both crystals are measured together by comparing speckles in the double-diffracted beam (‘sample’ data set) and the direct beam (‘reference’ data set). This mode is most helpful in characterizing DCMs, especially channel-cut crystals with restricted access to individual crystal surfaces.

**Figure 8:**
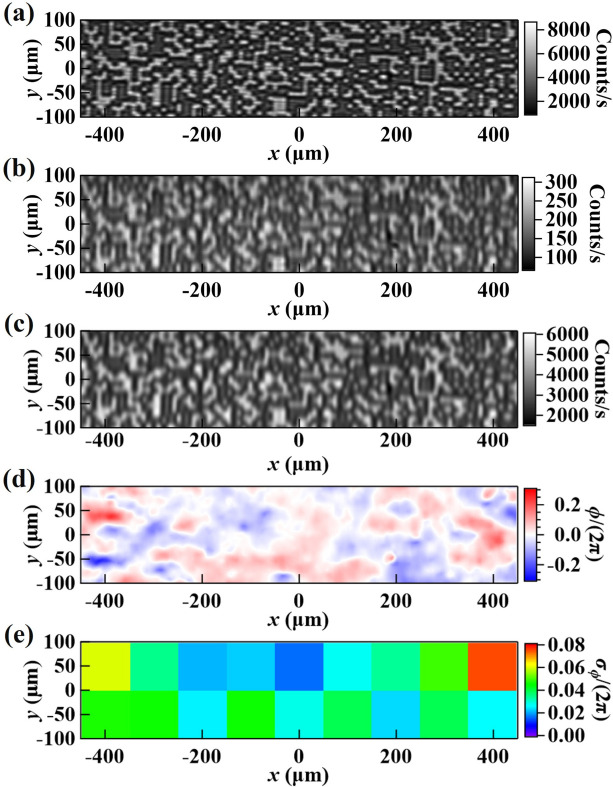
Speckle images in (*a*) the direct beam (‘reference’ data set) and (*b*) the diffracted beam (‘sample’ data set) generated by a double-diamond-crystal setup as shown in Fig. 2 ▸(*d*). (*c*) Gaussian-smoothed image of (*a*). (*d*) Reconstructed wavefront phase error, ϕ(*x*, *y*), introduced by the double-diamond-crystal reflections. (*e*) RMS phase errors, σ_ϕ_, within 100 m × 100 m (*x* × *y*) subareas extracted from (*d*).

Figures 8 ▸(*a*) and 8 ▸(*b*) show the speckle images in the direct and diffracted beams, respectively, in the double-diamond-crystal setup containing the same conditioning crystal and VB4. The intensity of the diffracted beam is much lower than that of the direct beam because of the narrower bandwidth of the diamond 400 diffraction (Δ*E*/*E* ≃ 10^−5^) compared with the direct beam bandwidth, which is defined by the 111 diffraction in the silicon monochromator (Δ*E*/*E* ≃ 1.4 × 10^−4^).

The speckle patterns from the coded mask can be identified from both images, but the contrast of speckles in the diffracted beam is much lower, especially in the vertical direction, because of the blurring *B*_*H*_ [equation (4)] due to the extinction effect. Blurring in the 400 Bragg reflection from diamond is *B*_400_ ≃ 7 µm, compared with *B*_111_ ≃ 1.5 µm for the 111 Bragg reflection from silicon crystals. As a result, applying the speckle-tracking algorithm directly to the data in Figs. 8 ▸(*a*) and 8 ▸(*b*) is problematic. Note that the wavefront phase information is stored only in the speckle displacement, not in the speckle contrast (which measures the beam transverse coherence). In order to extract the wavefront phase, a Gaussian smoothing was first applied to the direct beam image to blur it to a contrast level similar to that of the diffracted beam, as shown in Fig. 8 ▸(*c*). This step is necessary to improve the accuracy of phase reconstruction. Thus, Fig. 8 ▸(*c*) becomes the working ‘reference’ image. The wavefront phase error is then reconstructed using Figs. 8 ▸(*b*) and 8 ▸(*c*) as the ‘sample’ and ‘reference’ data sets, respectively; the results are shown in Fig. 8 ▸(*d*). The RMS phase error of the plotted area is σ_ϕ_ = 0.34 (or λ/19). A similar analysis was also carried out to determine that the best 100 µm × 100 µm beam subarea [Fig. 8 ▸(*e*)] has an RMS phase error of σ_ϕ_ = 0.10 (or λ/60) and a Strehl ratio of 0.989. Note that a 100 µm (*x*) × 100 µm (*y*) beam subarea corresponds to a 100 µm (*x*) × 200 µm (*l*) crystal surface area at the Bragg angle θ_B_ = 29.77°.

Finally, comparing the results of the absolute mode in Fig. 8 ▸(*d*) and the self-referencing mode in Fig. 6 ▸(*a*), we can note that the former has lost some of the high-spatial-frequency information during the smoothing step. Also, we expect the absolute mode to have a larger systematic error than the self-referencing mode because of the larger motion of both crystals and the additional detector motion. However, we can still observe a phase sensitivity beyond the λ/50 level, sufficient for evaluating the wavefront preservation of DCMs.

## 5. Conclusions

This work introduces a quantitative methodology to characterize crystal optics in X-ray Bragg diffraction using a state-of-the-art, coded-mask-based wavefront sensing technique. The method directly measures the wavefront phase error induced by the crystal optics and provides information on the Bragg-plane height error of the optics. These two measures are essential for evaluating the ability of high-quality crystal optics to preserve wavefronts and coherence, issues especially critical for the next-generation synchrotron light sources and X-ray free-electron lasers. The measured phase information can be used directly to simulate and predict beamline performance. A complete characterization of crystal optics can be provided by combining the proposed method with traditional topography-based techniques.

Different geometries have been examined. The double-crystal setup with the coded mask placed upstream is optimal for evaluating high-quality crystals. Two measurement types are introduced: the self-referencing single-crystal mode and the absolute double-crystal mode. The self-referencing mode gives the highest phase sensitivities, permitting study of crystals of ultra-high quality. The absolute double-crystal mode is more appropriate for characterizing double-crystal monochromators, especially channel-cut crystals, as a whole system. Both modes have been successfully demonstrated at the APS 1-BM beamline by characterization of the highest-quality silicon and diamond crystals. A systematic analysis procedure is also introduced to create a quantitative evaluation of the global quality of a crystal and its local wavefront error distribution. The proposed method will become a standard tool at the APS 1-BM beamline to assist crystal-related research and development and beamline optics characterization.
